# High-Quality Draft Genome Sequence of *Thermocrinis jamiesonii* GBS1^T^ Isolated from Great Boiling Spring, Nevada

**DOI:** 10.1128/genomeA.01112-16

**Published:** 2016-10-20

**Authors:** Rakesh Ganji, Senthil K. Murugapiran, John C. Ong, Namritha Manoharan, Marcel Huntemann, Alicia Clum, Manoj Pillay, Krishnaveni Palaniappan, Neha Varghese, Natalia Mikhailova, Dimitrios Stamatis, T. B. K. Reddy, Chew Yee Ngan, Chris Daum, Kecia Duffy, Nicole Shapiro, Victor Markowitz, Natalia Ivanova, Nikos Kyrpides, Tanja Woyke, Jeremy A. Dodsworth, Brian P. Hedlund

**Affiliations:** a School of Life Sciences, University of Nevada, Las Vegas, Las Vegas, Nevada, USA; b Department of Energy Joint Genome Institute, Walnut Creek, California, USA; c Department of Biology, California State University, San Bernardino, California, USA; d Nevada Institute of Personalized Medicine, University of Nevada, Las Vegas, Las Vegas, Nevada, USA

## Abstract

The draft genome of *Thermocrinis jamiesonii* GBS1^T^ is 1,315,625 bp in 10 contigs and encodes 1,463 predicted genes. The presence of *sox* genes and various glycoside hydrolases and the absence of uptake NiFe hydrogenases (*hyaB*) are consistent with a requirement for thiosulfate and suggest the ability to use carbohydrate polymers.

## GENOME ANNOUNCEMENT

Strain GBS1^T^ was isolated from the water column of Great Boiling Spring (GBS), Nevada, and described as a novel species, *Thermocrinis jamiesonii*, belonging to the family *Aquificaceae* (1). It is thermophilic, autotrophic, obligately microaerophilic, and grows chemolithoheterotrophically on peptone, casamino acids, or acetate with thiosulfate as the electron donor (1). It is different from other species of *Thermocrinis* in its use of thiosulfate as the sole electron donor and its high tolerance for NaCl (1).

The draft genome of strain GBS1^T^ was generated at the U.S. Department of Energy (DOE) Joint Genome Institute (JGI) using Illumina HiSeq 2000 sequencing technology yielding 18,071,694 filtered reads totaling 2.7 Gbp. Details of library construction and sequencing performed at JGI can be found at http://www.jgi.doe.gov. Filtered reads were assembled using Velvet (ver. 1.2.07) and Allpaths–LG (ver. r46652) (2, 3). The genome was annotated using Prodigal ver. 2.5 (4), as part of the JGI microbial annotation pipeline (5). The *T. jamiesonii* GBS1^T^ draft genome is 1,315,625 bp in 10 contigs, and encodes 1,463 predicted genes, including 1,415 protein-coding genes, 43 tRNA genes, and a single rRNA operon. Analysis of the genome for carbohydrate-active enzymes (CAZymes) (6) revealed 36 CAZymes, 6 of which are glycoside hydrolases (GHs) probably involved in degradation of chitodextrins/peptidoglycans (3 genes belonging to the GH23 family) and starch (GH13, GH57, GH77). These genes suggest GBS1^T^ might be capable of growth on some polymers, such as starch, as has been shown for *Thermocrinis minervae* (7). These cultivation and genomic data, along with *in situ* experiments, suggest some *Aquificales* to be mixotrophic or heterotrophic, rather than strictly autotrophic (8).

Consistent with the previous report (1), the GBS1^T^ genome encodes a *sox* gene cluster (*soxABXYZ*) required for thiosulfate oxidation (9). The genome lacks an NiFe hydrogenase (*hyaB*) and a canonical formate dehydrogenase (*fdhA*), which is consistent with the inability of GBS1^T^ to grow with H_2_ or formate as electron donors. However, the GBS water metagenome (JGI taxon identification number 2084038020; *hyaB*: GBSWBa_00119800; *fdhA*: GBSWBa_00059550) and a fraction of the *Thermocrinis* population in GBS has *hyaB* and/or *fdhA* (10). A variety of *Aquificales* fix CO_2_ via the reverse tricarboxylic acid (rTCA) cycle, including other *Thermocrinis* species, *Aquifex*, and *Hydrogenobacter* (11). The GBS1^T^ draft genome lacks 2-oxoglutarate-ferredoxin oxidoreductase, which is required for the rTCA cycle, but possesses other key enzymes, such as citryl-CoA lyase, citryl-CoA synthetase, and fumarate reductase (11). GBS1^T^ is capable of autotrophic growth, and the GBS water metagenome contains genes with high nucleotide identity to the *Thermocrinis albus* 2-oxoglutarate-ferredoxin oxidoreductase (GBSWBa_00110880), so it seems likely that GBS1^T^ possesses this gene but it is not present in the assembly. Though neither motility nor flagella was observed in cultures of GBS1^T^ (1), its genome has all the genes required for flagellar assembly, L rings, and P rings. The GBS1^T^ genome encodes capacity to synthesize C_16:0_, C_18:0_, and C_18:1_ω9c fatty acids, which were abundant cellular fatty acids along with the *Aquificales* C_20–22_ signature lipids (12) under standard growth conditions.

### Accession number(s).

The *T. jamiesonii* GBS1^T^ genome sequence is available in GenBank under the accession numbers JNIE01000001 to JNIE01000010. The data are also available from GenBank (NZ_JNIE00000000.1; GI: 657836485) and from the Joint Genome Institute (JGI) Integrated Microbial Genomes (IMG) system (2562617198) (13).
